# Knockdown of NRSF Alleviates Ischemic Brain Injury and Microvasculature Defects in Diabetic MCAO Mice

**DOI:** 10.3389/fneur.2022.869220

**Published:** 2022-05-13

**Authors:** Cheng-Feng He, Wen-Jiao Xue, Xiao-Die Xu, Jian-Tao Wang, Xin-Ru Wang, Yi Feng, Hou-Guang Zhou, Jing-Chun Guo

**Affiliations:** ^1^State Key Laboratory of Medical Neurobiology and MOE Frontiers Center for Brain Science, Department of Translational Neuroscience, Jing'an District Centre Hospital of Shanghai, Institutes of Brain Science, Fudan University, Shanghai, China; ^2^Department of Geriatric Neurology of Huashan Hospital, National Clinical Research Center for Aging and Medicine, Fudan University, Shanghai, China; ^3^State Key Laboratory of Medical Neurobiology, Department of Integrative Medicine and Neurobiology, School of Basic Medical Sciences, Institutes of Brain Science, Brain Science Collaborative Innovation Center, Fudan Institutes of Integrative Medicine, Fudan University, Shanghai, China

**Keywords:** NRSF/REST, diabetes, ischemic stroke, brain injury, NRP-1/VEGF, revascularization

## Abstract

Diabetes is one of the well-established risk factors of stroke and is associated with a poor outcome in patients with stroke. Previous studies have shown that the expression of neuron restrictive silencer factor (NRSF) is elevated in diabetes as well as ischemic stroke. However, the role of NRSF in regulating an outcome of diabetic ischemic stroke has not been completely understood. Here, we hypothesized that diabetes-induced NRSF elevation can aggravate brain injury and cognition impairment in ischemic stroke. The diabetic ischemic stroke mice model was established by 8 weeks of high-fat-diet feeding and 5 days of streptozotocin injection followed by 30 min of middle cerebral artery occlusion (MCAO). We found that diabetes enhanced the MCAO-induced elevation of NRSF in the hippocampus in accompany with an elevation of its corepressors, HDAC1, and mSin3A, and decrease of β-TrCP. By using histological/immunofluorescence staining and neurobehavioral testing, our results showed that the brain damage and learning/memory impairment were aggravated in diabetic ischemic mice but significantly attenuated after stereotaxic injection of NRSF-shRNA. Meanwhile, by performing whole-brain clearing with PEGASOS, microvascular reconstruction, western blotting, and ELISA, we found that NRSF-shRNA markedly alleviated the vasculature disorders and rescued the suppression of NRP-1, VEGF, and VEGFR2 in the hippocampus of diabetic ischemic mice. Therefore, our results demonstrated for the first time that the elevation of hippocampal NRSF plays an important role in alleviating brain injury and cognitive disabilities in diabetic ischemic mice, potentially *via* the reduction of NRP-1/VEGF signaling.

## Introduction

Diabetes mellitus (DM) is not only one of the major global health problems affecting hundreds and millions of people worldwide (1) but also one of the indispensable risk factors of stroke (2). It has been reported that diabetes coexists in ~40% of patients with ischemic stroke (3) and increases the mortality of stroke by 2-6 times (4). Mounting evidence has also suggested that diabetes is associated with worse stroke outcomes, such as the cognitive impairment and dementia (5–7). However, despite of the increasing evidence coming from the clinic, the biologic mechanism underlying diabetes and its negative stroke outcome is still unclear.

Neuron restrictive silencer factor (NRSF), also known as repressor element-1-silencing transcription factor (REST), is an important zinc-finger transcriptional repressor that binds a conserved 21 bp motif named neuron restrictive silencer element (NRSE) or repressor element 1 (RE-1) of target genes (8, 9). NRSF silences its target genes by recruiting corepressors, mSin3A, and CoREST to its N-terminal domain and C-terminal domain, respectively, and then forming a repressive transcriptional complex with histone deacetylases (HDACs) (10–12). Furthermore, two adjacent and distinct degron motifs in the NRSF-C-terminal domain allow NRSF protein to be regulated at the protein level by ubiquitination *via* β-TrCP-mediated proteasomal degradation (13, 14).

NRSF plays an important role in the development and progression of multiple neurological diseases, such as ischemic stroke, Alzheimer's disease, and Parkinson's disease (15–17). Increasing evidence indicated that neuronal expression of NRSF is upregulated during ischemic stroke and is related to neuronal death in hippocampal space (18–22). A study by Calderone et al. demonstrated that neuronal death following global ischemia was likely mediated by NRSF upregulation and its downstream suppression of GluR2, which can be rescued by the NRSF knockdown (18). Similarly, other studies found that NRSF can silence additional gene targets, such as OPRM1 (μ opioid receptor 1 or MOR-1) gene and miR-132 in neurons that were destined to death (19–21). Furthermore, it was pointed out that the upregulation of NRSF protein expression following global ischemia can also be mediated by its upstream effector Casein Kinase 1 (21), unveiling a complex but not thoroughly understood signaling network.

Interestingly, we previously found that neuronal expression of NRSF was upregulated in diabetic mice as well and contributed to diabetes-related neuronal injury and diabetic painful sensation (23, 24). Therefore, considering the increased risk and the poor outcome of stroke in diabetic patients and the elevation of NRSF shared in diabetic and ischemic mice models, we hypothesized that diabetes-induced NRSF elevation can aggravate brain injury and cognition impairment in ischemic stroke. Here, we aimed to assess alterations in the ischemic-induced neuronal injury, cerebral vascularization, and learning/memory abilities after administering AAV-NRSF shRNA into hippocampus to knockdown NRSF in diabetic ischemic mice and its downstream NRP-1/VEGF signaling pathway to further elucidate underlying mechanisms.

## Materials and Methods

### Animal Models and Grouping

Wild-type male C57BL/6 mice (6-8 weeks old) were purchased from Shanghai SLAC Laboratory Animal Co., Ltd (Shanghai, China), and housed in ventilated cages with free access to water and food in a light/dark cycle (12/12 h), temperature and humidity-controlled environment. All animal procedures were approved by the Institutional Animal Care and Use Committee of Shanghai Medical College of Fudan University. All efforts were made to minimize the suffering.

The diabetic/hyperglycemic mice model (DM) was established as described previously (25–27). In brief, the mice were fed adaptively with ordinary diet for 1 week, and then randomly grouped in the control group and the DM group. The DM mice were then fed with high-fat diet (FBSH, Shanghai, China) for 8 weeks, while the control mice with the ordinary diet for 8 weeks. After that, the mice were injected intraperitoneally with 40 mg/kg streptozotocin (STZ, Sigma-Aldrich, USA) or a citrate buffer for 5 days. One week later, those with fasting blood glucose higher than 11.1 mmol/L were defined as the successful diabetic/hyperglycemic mice (28–30).

**Figure 1:**
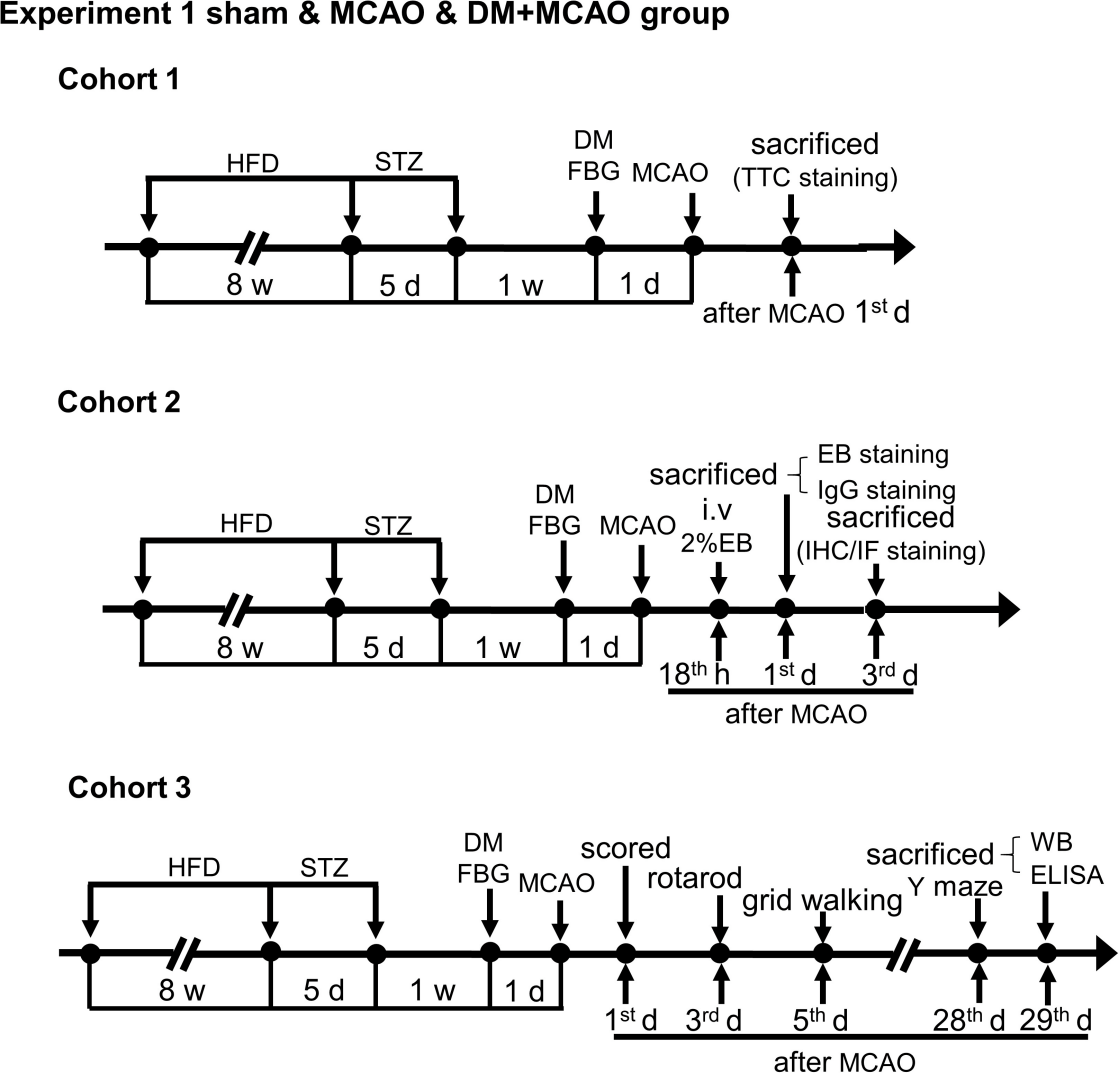
Schematic illustration of the experimental protocols of cohorts 1-3 in sham, MCAO, and DM + MCAO groups. The end points of detection of mice are also indicated. HFD, high-fat diet; STZ, streptozotocin; FBG, fasting blood glucose; MCAO, middle cerebral artery occlusion; i.v., intravenous injection; EB, Evans blue; w, week; d, day.

**Figure 2:**
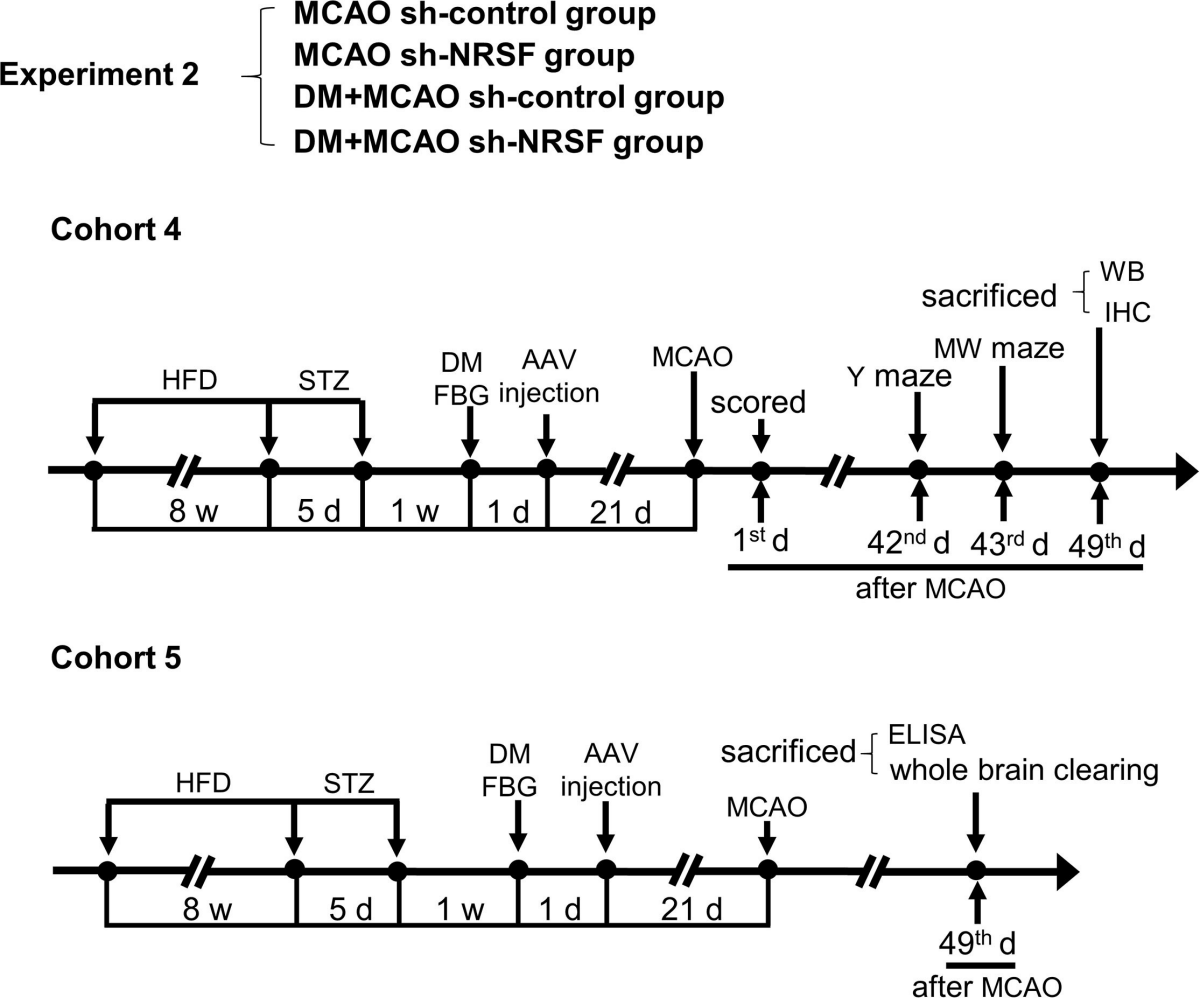
Schematic illustration of the experimental protocols of cohorts 4 and 5 in MCAO sh-control, MCAO sh-NRSF, DM + MCAO sh-control, and DM + MCAO sh-NRSF groups. The end points of detection of mice are also indicated. HFD, high-fat diet; STZ, streptozotocin; FBG, fasting blood glucose; MCAO, middle cerebral artery occlusion; w, week; d, day.

The transient middle cerebral artery occlusion (MCAO) model was performed essentially as previously described (31–33). Briefly, DM or control mice were kept on a feedback-controlled heating pad at 37 ± 0.5°C, anesthetized with 3% isoflurane in 30% O_2_ and 70% N_2_O for induction and 1.5% isoflurane for maintenance. A nylon monofilament coated with silicone rubber (CINONTECH, Beijing, China) was introduced into the internal carotid artery through the external carotid artery to occlude the middle cerebral artery (MCA) and removed 30 min later to allow reperfusion. Sham-operations were subject to the same procedure except for the MCA occlusion. Regional cerebral blood flow (rCBF) was monitored at the surface of ipsilateral parietal cortex during ischemia by using Laser Doppler Flowmetry (Periflux System 5000, Perimed) as previously described (31, 33). The mice were kept for further experiments and statistics analysis if blood flow perfusion dropped and maintained at ~20% of the preischemia baseline during ischemia. We performed MCAO surgery 1 week after the hyperglycemia onset in the Experiment 1, and 1 month thereafter in the Experiment 2 (Figures 1, 2), with a relatively longer diabetes duration.

### Knockdown of NRSF

The recombinant adeno-associated virus (AAV) shRNA expressing vectors containing NRSF-shRNA (5′-GCATGAAGTGACCCGACAT-3′) or non-specific control-shRNA (5′-TTCTCCGAACGTGTCACGT-3′) were constructed by Shanghai GeneChem using the pAKD-CMV-bGlobin-eGFP vector as previously reported (24). The mice were anesthetized with isoflurane, fixed in a stereotaxic apparatus, and 0.8-μl AAV solution was slowly injected unilaterally into the ipsilateral hippocampus (AP: −1.85 mm, ML: −1.60 mm, DV: −1.90 mm) at an infusion rate of 0.08 μl/min with a 5-μl capacity micro syringe (Gaoge, Shanghai, China), and then kept for 10 min after the injection. After suturing the scalp, the mice were placed in an incubator until recovery.

### Behavioral Tests

All the tests and analyses were performed according to the experiment schedule (Figures 1, 2) and blind to the observers.

#### Neurological Score

Neurological deficit was evaluated with a 28-point neurological score system as previously proposed by Clark et al. (34), which was performed 1 day after MCAO surgery.

#### Rotarod Test

Rotarod test (Ugo Basile, Italy) was used to assess motor coordination (35, 36) and performed at Day 3 after the MCAO surgery. The mice were pre-trained for three rounds with rotation speed at 5 rpm in the first round and accelerated from 5 to 40 rpm in 5 min in the second and third rounds. In the test sessions, the duration staying at the rotarod was recorded from three consecutive trials by 30-min interval. The final result was normalized with the baseline.

#### Grid Walking Test

The grid walking test was performed to evaluate motor function and performance of the contralateral forelimb at Day 5 after the MCAO surgery (37). The mice were pre-trained individually onto the grid for 5 min freely walking for 2 consecutive days. In the test session, the 5-min walking was recorded. The total steps and fault steps of the contralateral forelimb were counted until the total steps reached 100. The percentage of the forelimb fault was calculated as: the number of the fault steps/total steps (100) × 100%. The steps were defined as foot faults if the contralateral forelimb misses the grid and goes through the hole, or when the forelimb slips from the grid.

#### Y Maze

Y maze was used to evaluate spatial working memory by recording spontaneous alternation (38, 39), and performed at Day 28 (sham vs. MCAO vs. DM + MCAO group) and Day 42 (MCAO sh-control vs. MCAO sh-NRSF vs. DM + MCAO sh-control vs. DM + MCAO sh-NRSF group) after the MCAO surgery. The mice were placed in the center of the Y maze and freely explored three different arms for 10 min. The movement was recorded by EthoVision XT system (Noldus, Netherlands). The percentage of spontaneous alternation was calculated as: the number of spontaneous alternation/(total number of arm entries – 2) × 100%, entering three different arms in a row is considered a correct spontaneous alternation.

#### Morris Water Maze

Morris Water (MW) maze was used to assess spatial memory as previously reported (40, 41), which was performed at Day 43 after the MCAO surgery. The mice were gently placed from four varied entry points in the water facing the wall of the pool and trained to climb the hidden platform within 60 s for consecutive 5 days. The trial will end if mice climb the platform and stay at the platform for 15 s within 60 s. If mice failed to climb the platform, the experimenter will guide them to the platform and remain for 15 s. The mice were placed from the opposite side of the pool, an unacquainted entry point to find the disappeared platform, which was removed in advance on 6th day for a probe test. The mice were monitored and the data (swimming speed, times of platform crossing, percentage of time in the target quadrant) were recorded with EthoVision XT system (Noldus, Netherlands).

### Immunofluorescence Staining

Immunofluorescence staining was performed as described previously with minor alterations (42, 43). Briefly, the mice brain was coronally cut into 30-μm-thick sections by using frozen microtome (Leica, Germany) and stored in a cryoprotectant solution at −20°C. Frozen sections were permeabilized with 0.5% Triton X-100, blocked by 10% normal goat/donkey serum (Jackson, USA) and 0.3% Triton X-100 in PBS for 1 h, and incubated with specific primary antibodies (CD31, 1:200, goat, R&D systems; MAP2, 1:50, mouse, Abcam; NRSF, 1:200, rabbit, Biorbyt; PDGFR-β, 1:100, rabbit, Abcam; GFAP, 1:200, mouse, CST) at 4°C for 48 h. The primary antibodies were visualized by incubating with fluorophore-conjugated secondary antibodies as follows: Alexa Fluor 488 goat anti-mouse IgG (1:800, Jackson, USA); Alexa Fluor 488 donkey anti-goat IgG (1:800, Jackson, USA); Alexa Fluor 488 donkey anti-mouse IgG (1:800, Jackson, USA); Cy3 donkey anti-goat IgG (1:800, Jackson, USA); Cy3 donkey anti-rabbit IgG (1:800, Jackson, USA); Alexa Fluor Plus 594 goat anti-rabbit IgG (1:1,000, Invitrogen, USA). DAPI (1:500, CST, USA) were used for nuclei staining. Images were captured using a fluorescence microscope (Nikon, Japan) or a confocal microscope (Olympus, Japan) and analyzed by using Image J software. Pericyte coverage was quantified as the percentage of PDGFR-β^+^ CD31^+^ double-positive pericyte area/CD31^+^ area (total vascular area) × 100% as described previously (44). The vascular length and the area were measured using image analysis software Image J as described previously (45), and results were normalized to the mean value of the sham group.

### Histological Staining

TTC staining was performed 1 day after ischemia/reperfusion (46, 47). Freshly cut 1-mm-thick brain sections were stained with 2% 2,3,5-triphenyltetrazolium chloride (TTC, Sigma-Aldrich, USA) for 15 min at 37°C while protected from light, and then fixed in 4% PFA overnight and photographed. The infarcted area and the brain area in TTC-stained brain slices were measured using Image J software. The corrected percentage of infarction was calculated as the formula: infarction (%) = (the area of contralateral hemisphere – the area of non-infarcted ipsilateral hemisphere)/(2 ^*^ the area of contralateral hemisphere) ^*^100% (48).

Nissl staining was performed as previously reported (49, 50). The 30-μm frozen brain slices were mounted on glass slides coated with gelatin, dried for 48 h at 37°C, and then dehydrated and rehydrated with graded ethanol solutions (75, 85, 95, 100%), and stained with 0.25% cresyl violet (Sigma-Aldrich, USA) for 12 min. After that, sections were decolorized (95% ethanol containing 0.3% glacial acetic acid) and dehydrated in graded serried of ethanol. After being transparentized by xylene and sealed with neutral resin, the slices were observed under the light microscope (Nikon, Japan), and the number of surviving neurons per mm^2^ was counted.

Fluoro-Jade B (FJB) staining was performed in accordance with the manufacturer's instructions (Millipore, USA) to assess the neurodegeneration after MCAO.

BBB permeability was evaluated with an Evans blue (EB) assay and IgG staining as previously reported (51, 52). At 6 h before sacrificed and 18 h after MCAO, the mice were intravenously injected with 2% Evans blue (0.4 g/kg body weight; Sigma-Aldrich, USA) in sterile saline *via* femoral vein. For IgG staining, brain sections were incubated with IgG (Alexa Fluor 488 goat anti-mouse IgG, 1:500, Jackson, USA), and brain images were captured using the fluorescence microscope (Nikon, Japan).

### Western Blotting Analysis

Western blotting analysis was performed as reported previously with minor alterations (53). Total protein of brain tissue was extracted by tissue homogenization in a RIPA lysis buffer (EpiZyme, Shanghai, China) with protease inhibitor and phosphatase inhibitor cocktail (Rocher, Switzerland). The protein concentrations were assessed by the BCA Protein Assay Kit (Thermo, USA). Equal amounts of protein were loaded onto 7.5% SDS-PAGE gels to electrophoresis, and then transferred to the polyvinylidene difluoride membrane (PVDF; Millipore, USA), blocked with blocking solution (Epizyme, Shanghai, China), incubated with primary antibodies overnight at 4°C (NRSF, 1:1,000, Novus; HDAC1, 1:1,000, CST; mSin3A, 1:1,000, SAB; NRP-1, 1:1,000, Abcam; β-TrCP, 1:1,000, CST; β-actin, 1:20,000, Proteintech). Membranes were incubated with HRP-conjugated goat anti-rabbit and goat anti-mouse (1:10,000, CST) secondary antibodies after being washed in TBST. Proteins were visualized using ECL Kit (Epizyme, Shanghai, China). The gray value of protein bands was quantified with Image Lab software (Bio-Rad, USA) and normalized to β-actin.

### Enzyme-Linked Immunosorbent Assay

The proteins were extracted by tissue homogenization in 0.01 mmol/L PBS with protease inhibitor and phosphatase inhibitor cocktail (Rocher, Switzerland). Vascular endothelial cell growth (VEGF) and vascular endothelial cell growth factor receptor 2 (VEGFR2) levels in mice hippocampus were assessed by an ELISA kit (DiLab, Shanghai, China) according to the manufacturer's instructions.

### Whole-Brain Clearing

The polyethylene glycol-associated solvent system (PEGASOS) tissue-clearing method was performed as previously described (54, 55). The mice were intravenously injected with DyLight 649-labeled Tomato Lectin (1.25 mg/kg body weight; Vector Labs, USA) *via* femoral vein to label blood vessels after anesthetization. The brain was removed 5 min later and perfused with PBS containing 10 U/ml heparin sodium and 4% PFA, and then fixed in 4% PFA for 24 h, decolorized in 25% ethylenediamine for 2 days, and the solution was refreshed daily, and then washed with PBS for 30 min, delipidated with graded tert-Butanol (tB; 30, 50, 70%) solution, dehydrated with tB-PEG solution [tB: PEG-MMA-500 (polyethylene glycol methyl ether methacrylate average Mn500): ethylenediamine = 70:30:3, V/V], transparentized with benzyl benzoate (BB)-PEG solution (BB: PEG-MMA-500: ethylenediamine = 75:25:3, V/V) until the tissue turned transparent.

### Imaging and 3-Dimensional Reconstruction

Clearing brain images were acquired with the light-sheet microscopy (LS18, Nuohai, Shanghai, China). 3D reconstruction and analysis were performed with Imaris software version 9.7 (Bitplane, Switzerland). The Imaris Surface algorithm was used to semi-manually determine the identity of the hippocampus; the Filament algorithm was used for tracing blood vessels of hippocampus. Hippocampal volume, vascular volume, vascular length, vascular branch points were calculated by the Imaris software. The vascular volume density was quantified as hippocampal vascular volume/hippocampal volume. Vascular length density was quantified as hippocampal vascular length/hippocampal volume. Vascular branch points density was quantified as hippocampal vascular branch points/hippocampal volume.

### Statistical Analysis

Statistical analysis was performed using GraphPad Prism 7.0 software. Distributed continuous variables were expressed as mean ± SEM, and non-parametric data were presented as median (interquartile range). Data were compared with unpaired two-tailed Student's *T*-test or Mann–Whitney *U*-test between two groups, one-way ANOVA followed with Dunnett's or Tukey's multiple comparisons test among multiple groups, and two-way ANOVA followed by unpaired two-tailed Student's *T*-test between the two groups.

## Results

### Diabetes/Hyperglycemia Exacerbated MCAO-Induced Brain Damage and Behavioral Dysfunction

**Figure 3:**
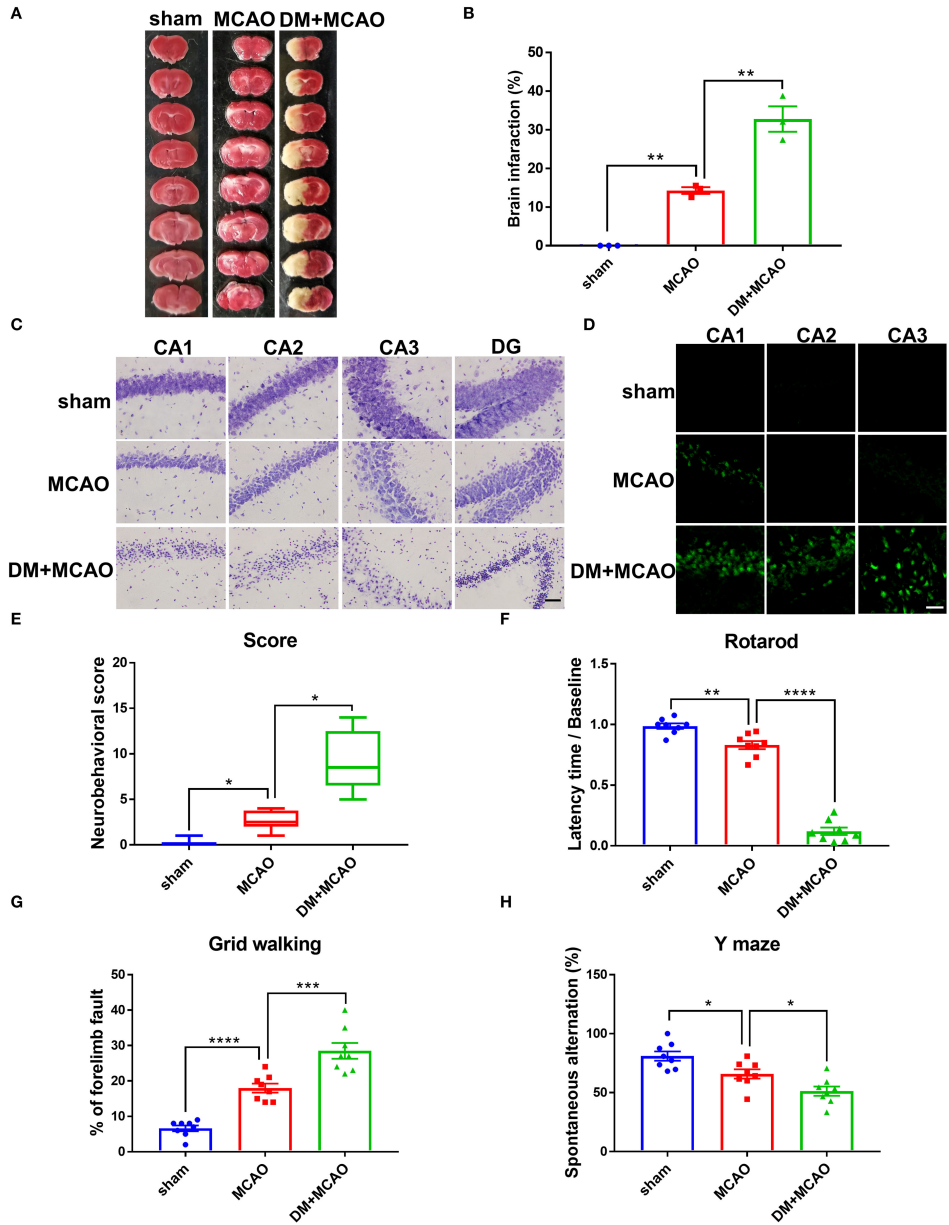
Diabetes greatly exacerbated MCAO-induced brain damage and neurobehavioral deficits. **(A)** Representative TTC staining images of mice in sham, MCAO, DM + MCAO group. TTC staining was performed 1 day after the MCAO surgery. **(B)** The statistical result of infarct sizes in all groups. *n* = 3 for each group. Data were shown as mean ± SEM (one-way ANOVA followed by Dunnett's multiple comparisons test). **(C)** Representative Nissl staining images of CA1, CA2, CA3, DG regions in mice. Scale bar = 100 μm. **(D)** Representative FJB staining images of CA1, CA2, CA3 regions in mice. Scale bar = 20 μm. **(E)** The statistical result of neurobehavioral scores of mice in all groups, which was performed 1 day after the MCAO surgery. *n* = 8 for each group. Data were shown as median (interquartile range, Kruskal-Wallis test followed by Dunn's multiple comparisons test). **(F,G)** The statistical results of rotarod test **(F)** and grid walking test **(G)** to evaluate motor function of mice in all groups. Rotarod test was performed at Day 3 after the MCAO surgery. The grid walking test was performed at Day 5 after the MCAO surgery. **(H)** The statistical result of Y maze of mice in all groups to evaluate spatial working memory, which was performed at Day 28 after the MCAO surgery. *n* = 8 for each group. Data were shown as mean ± SEM (one-way ANOVA followed by Dunnett's multiple comparisons test). *p < 0.05, **p < 0.01, ***p < 0.001, ****p < 0.0001.

To investigate whether diabetic/hyperglycemic conditions exacerbate the ischemic-induced brain injury, we performed MCAO operation at Day 1 after the DM mice model was successfully established. After TTC staining, our results confirmed that the diabetic/hyperglycemic ischemic mice (DM + MCAO) had significantly larger brain infarction when compared with the ischemia control (MCAO) mice (Figures 3A,B, *p* < 0.01). In the MCAO mice, the focal ischemia induced mild infarction mainly in ipsilateral cortex and striatum, while, in the DM + MCAO mice, the infarction mostly enlarged to the entire hemisphere, including hippocampus as well.

When we observed the ipsilateral hippocampus, data from Nissl staining and FJB staining showed that the DM + MCAO mice had significantly induced neuronal loss and increased FJB-positive cells (Figures 3C,D). In the MCAO mice, only sparse FJB-positive cells appeared in CA1 but was not evident in CA2 and CA3 areas. However, in the DM + MCAO mice, remarkably increased FJB-positive cells appeared in not only CA1 but also CA2 and CA3. These results suggested that diabetic ischemia induced much more drastic neural damage in the ipsilateral hippocampus than nondiabetic ischemia.

In addition, we assessed the neurobehavioral deficits in the mice. Previous studies found that motor functional disorders were more apparent in acute MCAO injury (56, 57). As shown in Figures 3E-G in our study, the DM + MCAO mice also exhibited more serious sensorimotor disorders. The neuro-deficit score notably increased in the MCAO mice and further upregulated in the DM + MCAO group 1 day after the MCAO (Figure 3E, *p* < 0.05, vs. the MCAO group). Rotarod test and grid walking test were performed at the 3rd and 5th day after surgery, respectively. Results showed that the mice in the DM + MCAO group spent less time on the rotating rod (Figure 3F, *p* < 0.0001, vs. the MCAO group) and performed more forelimb faults (Figure 3G, *p* < 0.001, vs. the MCAO group). Moreover, since diabetic ischemia induces greater damage in the ipsilateral hippocampus, we also assessed the hippocampus-dependent spatial learning and memory ability by performing Y maze test at the 28th day after the MCAO surgery. The percentage of spontaneous alternations was significantly decreased in the DM + MCAO group (Figure 3H, *p* < 0.05, vs. the MCAO group), indicating significantly impaired learning and memory function in the DM + MCAO mice.

**Figure 4:**
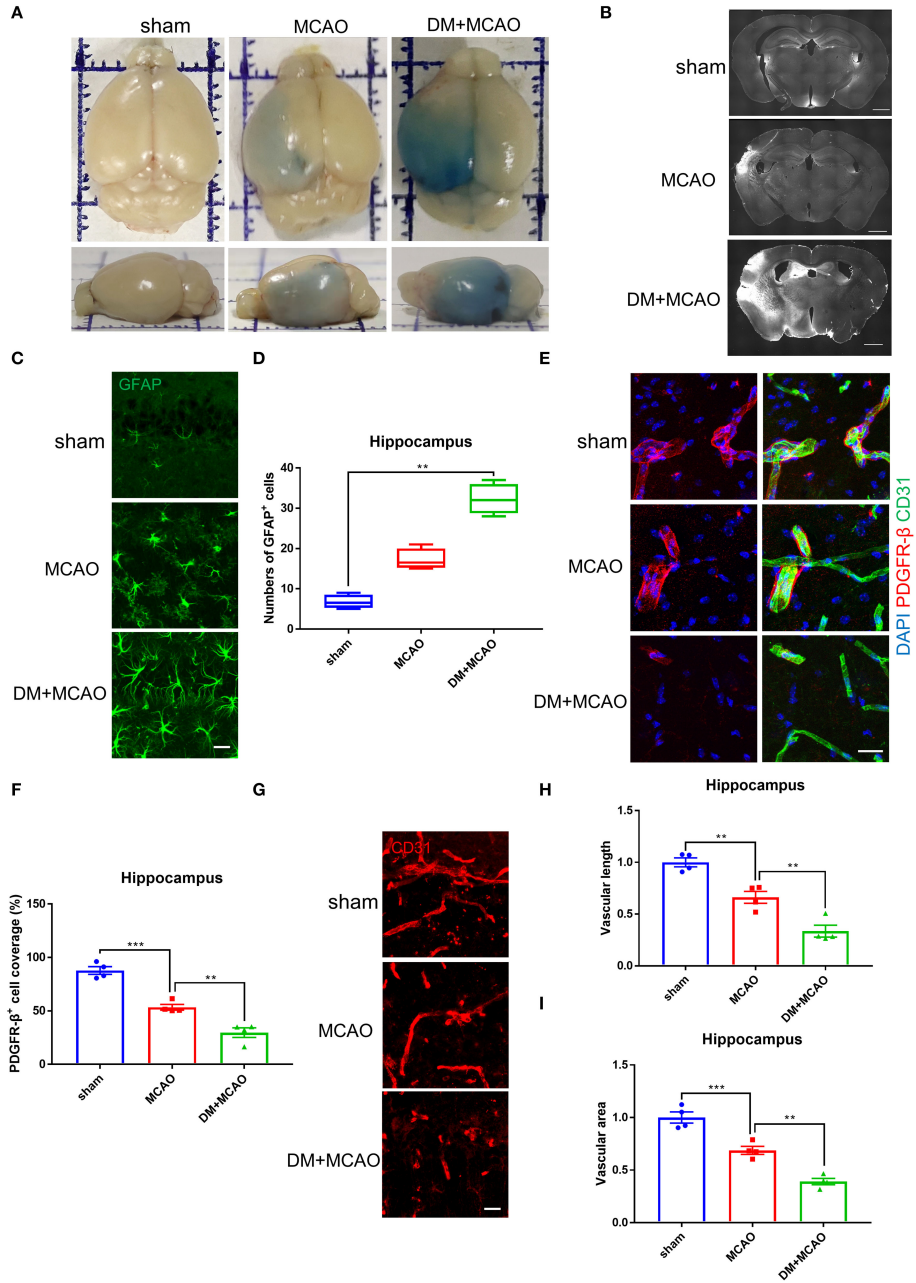
Diabetes greatly exacerbated MCAO-induced vasculature disorders. **(A)** Representative images of EB staining of mice in sham, MCAO, DM + MCAO group. Top view (above); side view (below). **(B)** Representative images of IgG staining. Scale bar = 1,000 μm. **(C)** Representative immunofluorescence staining images of GFAP-positive cells. Scale bar = 20 μm. **(D)** Quantification of GFAP-immunoreactivity-positive cells per visual field in hippocampus of mice. *n* = 4 for each group. Data were shown as median (interquartile range, Kruskal-Wallis test followed by Dunn's multiple comparisons test). **(E)** Representative immunofluorescence staining images of PDGFR-β-positive pericytes (red) coverage on CD31-positive blood vessels (green). Scale bar = 20 μm. **(F)** Quantification of PDGFR-β-positive pericytes coverage on CD31-positive blood vessels in ipsilateral hippocampus. *n* = 4 for each group. Data were shown as mean ± SEM (one-way ANOVA followed by Dunnett's multiple comparisons test). **(G)** Representative immunofluorescence staining images of CD31-positive blood vessels in ipsilateral hippocampus. Scale bar = 20 μm. **(H)** Quantification of CD31-positive microvascular length in hippocampus. The results were normalized to the mean value of the sham group. *n* = 4 for each group. Data were shown as mean ± SEM (one-way ANOVA followed by Dunnett's multiple comparisons test). **(I)** Quantification of CD31-positive microvascular density in hippocampus. The results were normalized to the mean value of the sham group. *n* = 4 for each group. Data were shown as mean ± SEM (one-way ANOVA followed by Dunnett's multiple comparisons test). **p < 0.01, ***p < 0.001.

Cerebral ischemia leads to not only neuronal injury but also vasculature disorders, we further detected the blood-brain barrier (BBB) permeability by using the EB assay and IgG staining. As shown in Figures 4A,B, the EB leakages and the IgG-fluorescent area were greatly increased in ischemic hemisphere of the DM + MCAO mice compared with that of the MCAO mice. In consistent with the severer hippocampal neuronal damage in the DM + MCAO mice, the BBB leakage is also obvious in the hippocampus of the DM + MCAO mice. Next, we observed abnormal alterations of the BBB components. GFAP is the specific marker of astrocytes. As shown in Figures 4C,D, GFAP^+^ cells appeared apparently swollen, and the number of GFAP^+^ cells increased significantly in ipsilateral hippocampus of the DM + MCAO mice when compared with the sham mice (*p* < 0.01), suggesting that activated astrocytes increased in the DM + MCAO mice. Studies have found that pericyte coverage is negatively correlated with vascular maturation and BBB permeability (58–60), we further detected pericyte coverage on the blood vessels after DM + MCAO application. PDGFR-β is one of the specific markers of pericyte. As shown in Figures 4E,F, the PDGFR-β^+^ coverage on blood vessels was substantially decreased in the ipsilateral hippocampus of the DM + MCAO mice (*p* < 0.01, vs. the MCAO group). Moreover, our data showed that, in the ipsilateral hippocampus of the DM + MACO mice, CD31-labeled brain vascular length (Figures 4G,H, *p* < 0.01, vs. the MCAO group) and density (Figures 4G,I, *p* < 0.01, vs. the MCAO group) were remarkably reduced. These results suggested that diabetes/hyperglycemia worsened ischemia-induced BBB integrity and BBB vasculature disorders.

Therefore, the above results collectively demonstrated that the diabetic/hyperglycemic condition could further worsen cerebral ischemia-induced neuronal damage and vasculature disorders and deteriorate neurobehavioral disabilities.

### Diabetes/Hyperglycemia Enhanced MCAO-Induced Elevation of NRSF and Its Corepressors While Decreased the β-TrCP Level

**Figure 5:**
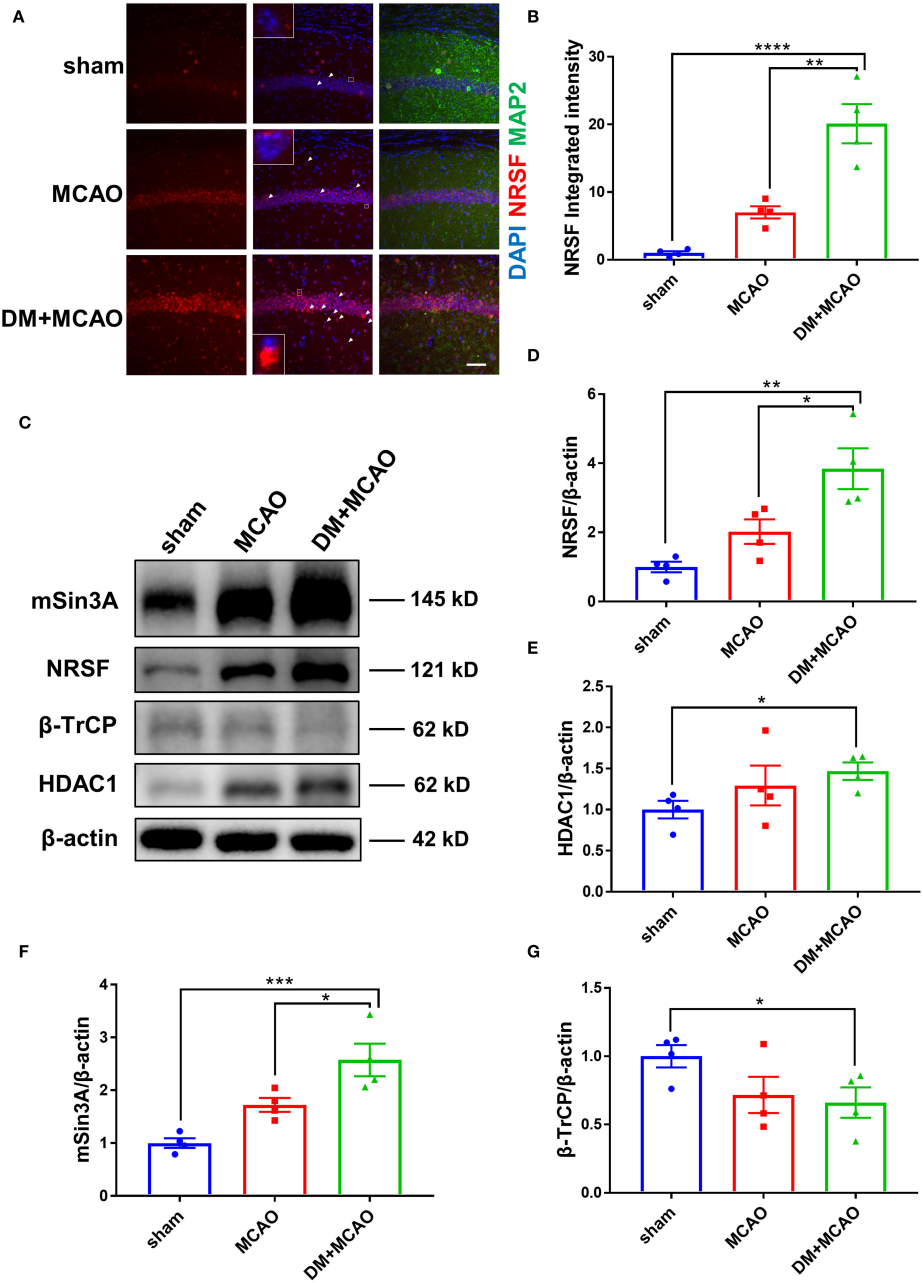
Diabetes enhanced MCAO-induced elevation of NRSF and its corepressors and decreased the expression of β-TrCP in hippocampus of mice. **(A)** Immunofluorescence staining of NRSF (red); cell nuclei were stained with DAPI (blue); neurons were stained with MAP2 (green). White arrows indicate representative cells with extranuclear NRSF. The inset is the local magnified image of representative cell. Scale bar = 50 μm. **(B)** The statistical results of integrated fluorescent intensity of hippocampal NRSF. Integrated fluorescent intensity was normalized to the mean value of the sham group, *n* = 4 for each group. Data were shown as mean ± SEM (one-way ANOVA followed by Tukey's multiple comparisons test). **(C)** Representative western blot images of NRSF, HDAC1, mSin3A, β-TrCP in hippocampus of all groups. **(D)** Quantification graphs of NRSF in hippocampus of all groups. *n* = 4 for each group. Data were shown as mean ± SEM (one-way ANOVA followed by Tukey's multiple comparisons test). **(E)** Quantification graphs of HDAC1 in hippocampus of all groups. *n* = 4 for each group. Data were shown as mean ± SEM (unpaired two-tailed Student's *T*-test). **(F)** Quantification graphs of mSin3A in hippocampus of all groups. *n* = 4 for each group. Data were shown as mean ± SEM (one-way ANOVA followed by Tukey's multiple comparisons test). **(G)** Quantification graphs of β-TrCP in hippocampus of all groups. *n* = 4 for each group. Data were shown as mean ± SEM (unpaired two-tailed Student's *T*-test).*p < 0.05, **p < 0.01, ***p < 0.001, ****p < 0.0001.

We previously reported that NRSF elevation participates in neuronal injury under hyperglycemia environment (24), and then we detected NRSF levels in the MCAO and DM + MCAO mice by using the immunofluorescent staining in the present study. As shown in Figure 5A, the NRSF fluorescent particle was obviously increased in the hippocampus of the MCAO mice, and further enhanced in the DM + MCAO mice. Observing the subcellular distribution of NRSF, we further found that, in some neurons, the NRSF particles only localized in the nucleus, while, in other neurons, the NRSF particles localized both in the nucleus and cytoplasm or almost exclusively clustered in the extranuclear area (see white arrows in Figure 5A). Statistical analysis revealed that, in the hippocampus of the DM + MCAO mice, the integrated fluorescence intensity of NRSF was significantly enhanced (Figure 5B, *p* < 0.01, vs. the MCAO group).

Western blotting analysis showed accordant results that the NRSF protein level was markedly increased in the hippocampus of the DM + MCAO mice (Figures 5C,D, *p* < 0.05, vs. the MCAO group). Interestingly, we found that the expression of NRSF cofactors, HDAC1, and mSin3A also increased significantly in the DM + MCAO mice, following the same pattern as that of NRSF (Figures 5C,E,F). Therefore, these results demonstrated that diabetes/hyperglycemia enhanced MCAO-induced elevation of NRSF and its corepressors, which indicated the deepened activation of NRSF in the DM + MCAO mice.

β-TrCP is a well-known E3 ubiquitin ligase, which could mediate proteasomal degradation that regulates the expression of NRSF by ubiquitination (21). Thus, we detected the hippocampal β-TrCP level in the MCAO and DM + MCAO mice. Consequently, the expression of β-TrCP was reduced in the MCAO group and the DM + MCAO group (Figures 5C,G, *p* < 0.05, vs. the sham group).

### Knockdown of NRSF Ameliorated the Diabetes-Worsened Ischemic Brain Damage

**Figure 6:**
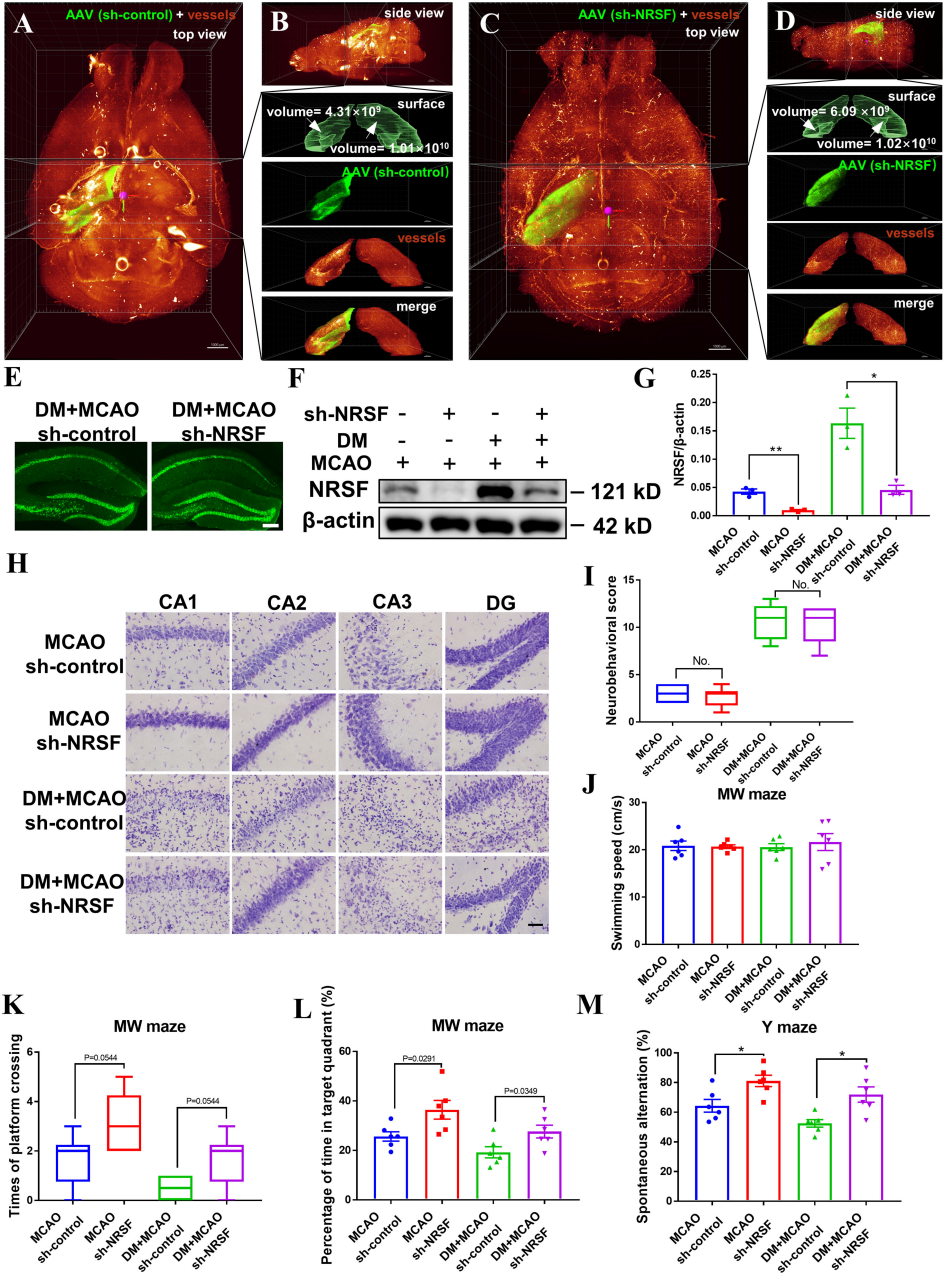
The hippocampal NRSF knockdown ameliorated the diabetes-worsened ischemic brain damage and memory deficit. **(A-D)** Images of the whole brain of the DM + MCAO mice by clearing with PEGASOS and 3D reconstruction. GFP-tagged AAV-shRNA (green), tomato lectin-labeled vasculature (red). Lower right was the local magnified image of hippocampus, upper to lower: Imaris Surface algorithm simulated the hippocampus surface by semi-manually tracking; the signal of the GFP-tagged AAV shRNA (green) inner hippocampus surface; the signal of the tomato lectin-labeled vasculature (red) inner hippocampus surface; the signal of GFP-tagged AAV shRNA (green) merged with tomato lectin-labeled vasculature (red). **(A)** The top view of the whole brain in the DM + MCAO sh-control mice. **(B)** The side view of the whole brain in the DM + MCAO sh-control mice. **(C)** The top view of the whole brain in the DM + MCAO sh-NRSF mice. **(D)** The side view of the whole brain in the DM + MCAO sh-NRSF mice. Scale bar = 1,000 μm. **(E)** Schematic images of GFP expression in the ipsilateral hippocampus of sh-control and sh-NRSF mice. Scale bar = 200 μm. **(F)** Representative western blot image for NRSF in ipsilateral hippocampus. **(G)** Protein quantification of NRSF in the hippocampus of mice. β-actin was used as loading controls. n = 3 for each group. Data were shown as mean ± SEM (two-way ANOVA, Student's *T-*test). **(H)** Representative Nissl staining images of CA1, CA2, CA3, DG regions. Scale bar = 100 μm. **(I)** The statistical result of neurobehavioral scores of mice in sh-control and sh-NRSF groups, which was performed 1 day after the MCAO surgery. *n* = 6 for each group. Data were shown as median (an interquartile range, Mann–Whitney test). **(J)** The statistical results of swimming speed in Morris water maze test, which was performed at Day 43 after the MCAO surgery. n = 6 for each group. Data were shown as mean ± SEM (two-way ANOVA, Student's *T*-test). **(K)** The statistical results of times of platform crossing in Morris water maze test. *n* = 6 for each group. Data were shown as median (an interquartile range, Mann–Whitney test). **(L)** The statistical results of percentage of time in the target quadrant in Morris water maze test. *n* = 6 for each group. Data were shown as mean ± SEM (two-way ANOVA, Student's T-test). **(M)** The statistical result of percentage of spontaneous alternation in Y maze test, which was performed at Day 42 after the MCAO surgery. n = 6 for each group. Data were shown as mean ± SEM (two-way ANOVA, Student's *T*-test). *p < 0.025, **p < 0.005.

Patients with stroke with chronic diabetes commonly present deficits in learning/memory abilities, which are closely related to the functions of hippocampus (61–66). Since we observed that the neural and vascular injuries increased and the NRSF is elevated in the hippocampus of the DM + MCAO mice, we investigated whether the enhanced hippocampal NRSF participates in diabetic ischemia-worsened brain damage and learning/memory abilities. Here, we intra-hippocampally injected NRSF shRNA (sh-NRSF) in the DM + MCAO mice to knockdown the expression of NRSF at 1 week after the hyperglycemia onset and performed the MCAO surgery at 1 month after the hyperglycemia onset. As shown in Figures 6A-G, the green fluorescence of sh-NRSF precisely localized in the ipsilateral hippocampus and effectively knocked down the NRSF expression, which coincides with our previous study (24). By whole-brain clearing, we semi-manually determined the identity of the hippocampus and assessed hippocampal volume by Imaris Surface algorithm. As shown in Figures 6B,D, although the contralateral hippocampus volume of the two groups was approximately equal (DM + MCAO sh-control: 1.01 × 10^10^ mm^3^; DM + MCAO sh-NRSF: 1.02 × 10^10^ mm^3^), the volume of ipsilateral ischemic hippocampus in the DM + MCAO sh-NRSF group was about 1.5 times larger than that of the DM + MCAO sh-control group (DM + MCAO sh-control: 4.31 × 10^9^ mm^3^; DM + MCAO sh-NRSF: 6.09 × 10^9^ mm^3^), which suggested that the knockdown of NRSF might ameliorate diabetic ischemic hippocampal atrophy. Meanwhile, the Nissl staining confirmed that the NRSF knockdown rescued neuronal loss, shrinks, and deformations in hippocampus of the MCAO and DM + MCAO mice, and the number of surviving neurons was also increased after the NRSF knockdown (Figure 6H, Supplementary Figure 1).

To confirm that this beneficial effect is not due to the surgery-induced stroke severity differences between sh-control and sh-NRSF groups, we also assessed the sensorimotor deficits 1 day after the MCAO surgery in these mice. As shown in Figure 6I, no significant differences were observed between the two groups in the MCAO mice or in the DM + MCAO mice. Previous studies have found that the sensorimotor deficits of the animals surviving up to 24 h after MCAO are positively correlated with the infarct size in the brain (67), thus, these results indicated that the hippocampally injection does not affect the acute MCAO severity in the sh-control and sh-NRSF mice. In addition, we evaluated hippocampus-dependent spatial learning and memory ability in the mice after the knockdown of NRSF by Y maze and MW maze. The Y maze test was performed at 42nd day, and the MW maze test was performed 43rd-49th days after MCAO. Our data showed that there was no significant difference in swimming speed among the four groups (Figure 6J). However, the times of platform crossing (Figure 6K) and percentage of time in the target quadrant (Figure 6L) were increased in the sh-NRSF mice when compared with the sh-control mice. The percentage of spontaneous alternation was increased as well in the MCAO sh-NRSF and DM + MCAO sh-NRSF mice (Figure 6M). These results indicated that the NRSF knockdown in hippocampus rescued spatial memory of the mice in the MCAO sh-NRSF and DM + MCAO sh-NRSF groups.

Therefore, the above results demonstrated that the NRSF knockdown attenuates neuronal injury in hippocampus, which is induced by diabetic ischemic stroke.

### Knockdown of NRSF Alleviated the Diabetes-Worsened Ischemic Vasculature Disorder and Upregulated the Expression of NRP-1/VEGF/VEGFR2

**Figure 7:**
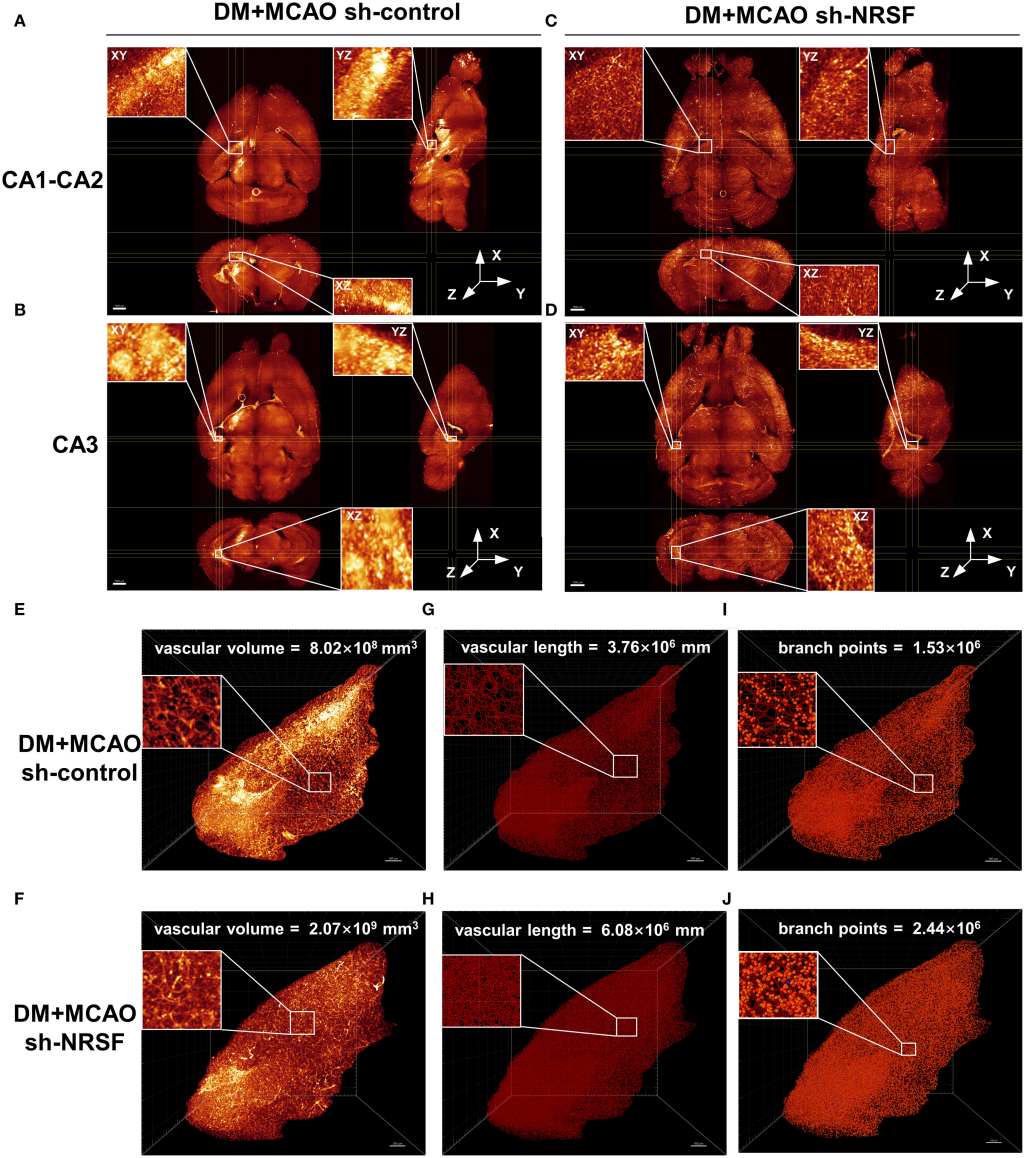
The NRSF knockdown ameliorated the diabetes-worsened ischemic vasculature disorder. **(A-D)** Representative images of the CA1-CA2 **(A,B)** and CA3 **(C,D)** regions in mice of DM + MCAO sh-control and DM + MCAO sh-NRSF groups. Top left: the XY orthogonal view; bottom left: the XZ orthogonal view; right: the YZ orthogonal view. Scale bar = 1,000 μm. **(E-J)** Images of ischemic ipsilateral hippocampal vascular volume **(E,F)**, length **(G,H)**, and branch points **(I,J)** in mice of DM + MCAO sh-control and DM + MCAO sh-NRSF groups were calculated with Imaris Filament algorithm. The inset is the local magnified image. Scale bar = 300 μm.

By performing whole brain clearing with PEGASOS, we investigated whether the knockdown of NRSF could also rescue hippocampal vasculature impairment in the DM + MCAO mice. As shown in Figures 7A,B, the ipsilateral ischemic hippocampus of the DM + MCAO sh-control mice exhibited abnormal fluorescence patterns in the CA1-CA2 and CA3 regions. In hippocampus of the DM + MCAO sh-NRSF mice, the fluorescence distribution was more uniform (Figures 7C,D). Next, we assessed the ipsilateral ischemic hippocampal vascular volume, length, and branch points by Imaris Filament algorithm. Our data showed that the vascular volume (Figures 7E,F, 8.02 × 10^8^ mm^3^ in sh-control vs. 2.07 × 10^9^ mm^3^ in sh-NRSF), vascular length (Figures 7G,H, 3.76 × 10^6^ mm in sh-control vs. 6.08 × 10^8^ mm in sh-NRSF), vascular branch points (Figures 7I,J, 1.53 × 10^6^ in sh-control vs. 2.44 × 10^6^ in sh-NRSF) were all greatly increased in hippocampus of the DM + MCAO mice after the NRSF knockdown. Meanwhile, we also calculated the density of vascular volume, length, and branch points in ipsilateral hippocampus and found that the densities (for vascular volume: 0.34 in sh-NRSF vs. 0.19 in sh-control; for vascular length: 9.98 × 10^−4^ in sh-NRSF vs. 8.72 × 10^−4^ in sh-control; for vascular branch points: 4.00 × 10^−4^ in sh-NRSF vs. 3.54 × 10^−4^ in sh-control) were substantially improved as well.

**Figure 8:**
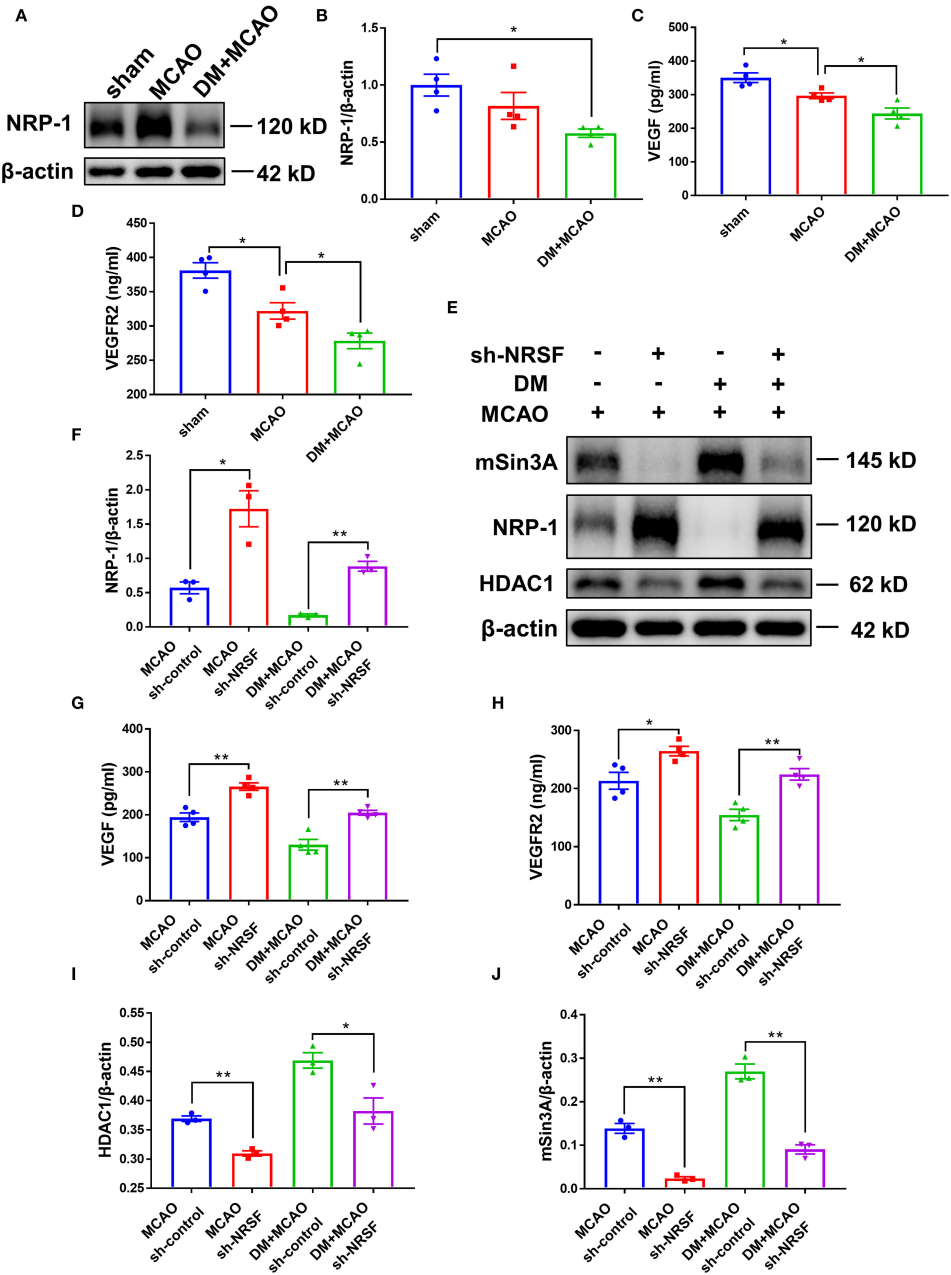
The knockdown of NRSF upregulated the expression of NRP-1/VEGF/VEGFR2. **(A)** Representative western blot images of NRP-1 in the ipsilateral hippocampus of sham, MCAO, and DM + MCAO mice. **(B)** Quantification graphs of NRP-1 in the hippocampus of mice. β-actin was used as loading controls. The results were normalized to the mean value of the sham group. *n* = 4 for each group. Data were shown as mean ± SEM (one-way ANOVA followed by Tukey's multiple comparisons test).*p < 0.05. **(C,D)** Protein quantification of VEGF and VEGFR2 by ELISA. *n* = 4 for each group. Data were shown as mean ± SEM (one-way ANOVA, followed by Dunnett's multiple comparisons test). *p < 0.05. **(E)** Representative western blots images of HDAC1, mSin3A, NRP-1 in the hippocampus of MCAO sh-control, MCAO sh-NRSF, DM + MCAO sh-control, and DM + MCAO sh-NRSF groups. **(F)** Protein quantification of NRP-1 in the hippocampus of MCAO sh-control, MCAO sh-NRSF, DM + MCAO sh-control, and DM + MCAO sh-NRSF group. β-actin was used as loading controls. *n* = 3 for each group. Data were shown as mean ± SEM (two-way ANOVA, Student's *T*-test). *p < 0.025, **p < 0.005. **(G,H)** Protein quantification of VEGF and VEGFR2 in the hippocampus of mice by ELISA. *n* = 4 for each group. Data were shown as mean ± SEM (two-way ANOVA, Student's *T*-test).*p < 0.025, **p < 0.005. **(I,J)** Protein quantification of HDAC1 **(I)**, mSin3A **(J)** in the hippocampus of mice. β-actin was used as loading controls. *n* = 3 for each group. Data were shown as mean ± SEM (two-way ANOVA, Student's *T*-test). *p < 0.025, **p < 0.005.

The above results suggested that the NRSF knockdown protected microvasculature against diabetic ischemic injury. To elucidate the possible mechanisms underlying, we detected Neuropilin-1 (NRP-1)/VEGF signaling by using western blotting analysis and ELISA. As shown in Figures 8A,B, the NRP-1 level further decreased in the DM + MCAO mice (*p* < 0.05, vs. the sham group). The expression of VEGF and VEGFR2 was detected by ELISA. The results showed that their expression decreased as well in the DM + MCAO mice when compared with the MCAO mice (Figures 8C,D, both *p* < 0.05 vs. the MCAO group). After the knockdown of NRSF, NRP-1 levels significantly increased in both the MCAO and DM + MCAO mice (Figures 8E,F). The findings of ELISA showed that both VEGF and VEGFR2 were significantly upregulated after the NRSF knockdown (Figures 8G,H). These results suggested that the NRSF knockdown ameliorates the abnormal hippocampal vascularization probably by upregulation of NRP-1/VEGF signaling. Moreover, we found that corepressors HDAC1 and mSin3A levels were remarkably reduced after the NRSF-knockdown (Figures 8E,I,J) reconfirmed the inactivation of NRSF.

## Discussion

In the present study, we found that the NRSF is further elevated in the hippocampus of the diabetic ischemic mice and participates in the brain injury aggravation and worsening of learning/memory functions. By using sh-NRSF injection and 3-dimentional visualization, we also illuminated the correlation between the NRSF knockdown and revascularization in diabetic ischemic hippocampus. Moreover, we declared that the decreased β-TrCP is possibly involved in the NRSF activation and that NRP-1/VEGF signaling may offer a downstream pathway of NRSF in deteriorating brain damage upon diabetic ischemic conditions. To our knowledge, this is the first study to investigate the role of NRSF in diabetic ischemic brain injury, and observed microvasculature in the hippocampus of the intact diabetic ischemic mice by using PEGASOS brain clearing and 3-dimensional reconstruction for the first time. Our findings highlight that NRSF elevation is one of the important events during the process of hippocampus-dependent learning/memory impairment in diabetic ischemia.

Diabetes is well-recognized as an important risk factor in neurovascular disease. Studies found that patients with diabetes had higher incidence of ischemic stroke than hemorrhagic stroke (68). Previous studies reported that chronic hyperglycemia and acute hyperglycemic stress could drive the pathological processes of ischemic injury and is associated with poorer outcomes, such as increased mortality and impaired recovery, in patients with stroke (5, 69, 70). In accordance with these clinical observations, here in our study, we found that diabetes worsened ischemic neuronal injury, which is confirmed by the enlarged ischemic infarction and graver neuronal loss and degeneration in mice brain. Moreover, in consistent with the fact that patients with stroke with chronic diabetes are prone to learning/memory disabilities (61–64), we also found that the diabetic ischemic mice had serious injury in hippocampus, an area remote to the site of injury, which was associated with greater impairment of memory behaviors. These findings are in accordance with previously reported animal studies (71, 72), which further confirmed that the comorbidity of diabetes with cerebral ischemia induces more harmful pathogenesis in hippocampus.

Studies reported that brain NRSF is elevated after cerebral ischemia (18–21). We also found that diabetes induces NRSF expression elevation in our previous studies (23, 24). In this study, we clarified that the expression of NRSF and its corepressors are further elevated in hippocampus in diabetic ischemia compared with non-diabetic ischemia. Given that NRSF and its corepressors can form restrictive complex to silence a variety of neural genes (11, 73), the elevation of NRSF, and its corepressors, as observed in this study, indicate that a wide-scale change in downstream protein transcription might have been triggered during diabetic ischemic neuronal injury. In the present study, our data showed that the knockdown of hippocampal NRSF did not affect the acute post-ischemic sensorimotor deficits after the MCAO surgery, but greatly attenuated diabetic ischemia-induced neuronal injury in hippocampus and improved learning/memory abilities, thus demonstrated that NRSF elevation in hippocampus plays an important role in the deterioration of ischemia-induced injury and hippocampus-dependent learning/memory impairment under diabetic/hyperglycemia condition.

Accumulated evidence demonstrated that BBB leakage and microvasculature disorder induced by cerebral ischemia/reperfusion can lead to severely impaired ability of neural repair and regeneration (74–76). In this study, we found that cerebral ischemia induced more drastic BBB leakage when diabetes coexisted. The BBB is the most important barrier involved in the neurovascular unit. It is composed mainly of brain endothelial cells, astrocyte end-feet, microglia, oligodendrocytes, and pericytes, integrated by tight junctions, adherent junctions, and gap junctions between the endothelial cleft. These cells strictly control the specific substances entering or clearing between the circulation and the brain, and maintain the homeostasis of brain metabolite activities (77–79). In the present study, our data showed that, the DM + MCAO mice had significantly increased number of activated astrocytes, reduced coverage of pericytes on microvasculature, and impaired microvasculature with less vascular length and density when compared with the MCAO mice. These data collectively demonstrated that diabetic ischemia leads to the more aggravated microvasculature disorder and BBB permeability.

In this paper, we performed the polyethylene glycol (PEG)-associated solvent system (PEGASOS) method for brain clearing and evaluating the impaired microvasculature. Studies have reported that the PEGASOS method renders nearly all types of tissues transparent and offers better fluorescence preservation than other solvent-based clearing methods (54, 80). Here, we imaged 3-D visualization of intact mouse brain microvasculature and detected vascular-related parameters, including branching, length, and volume in the hippocampus. In the study, we observed 3 mice in each group. Unfortunately, bubbles were found inside the brain of one DM + MCAO sh-NRSF mouse during brain clearing. Therefore, we failed to obtain correct data from this mouse and conduct statistical analysis. However, based on data from other mice, a consistent trend was shown between groups as depicted in Figure 7. Our data showed for the first time that diabetic ischemia-induced alterations of vascular plasticity were remodeled by the reduction of NRSF expression. Compared to the sh-control DM + MCAO mice, the vascular volume, length, and branching increased in the sh-NRSF DM + MCAO mice. These results indicated that NRSF elevation may participate in the impaired microvasculature under diabetic cerebral ischemia, which is partly responsible for worsened neuronal injury.

Previous studies have reported that NRSF is a substrate for the skp1-cullin-F box family of ubiquitin ligases E3s β-TrCP. The binding of β-TrCP to NRSF enables the ubiquitin-based proteasomal degradation process of NRSF (13). Overexpression of β-TrCP results in decreased protein stability and abundance of NRSF (21). Here, in the present study, we found that β-TrCP levels are lessened after MCAO and DM + MCAO application. Thus, the NRSF elevation in the diabetic ischemic mice might be related with the reduced β-TrCP-mediated proteasomal degradation. However, the stability and abundance of NRSF are also regulated by other mechanisms, such as de-ubiquitination, phosphorylation, and transcription (21, 22, 81), whether these modifications are responsible for the activation of NRSF under diabetic ischemia needs further investigation.

Neuropilin-1 (NRP-1) is a non-tyrosine kinase transmembrane protein mainly expressed in neurons and endothelial cells (82, 83). Studies have found that NRP-1 serves as a coreceptor for the VEGF165, forms tertiary complex with VEGFR-2, and enhances VEGF activity by promoting angiogenesis (84). It has been reported that NRP-1 upregulation decreased hypoxia-induced apoptosis about three-fold, while Nrp1-deficient mice are embryonic lethal and exhibited both neuronal and vascular defects (85–87). Nrp1 contained NRSE in the promoter region between −173 and −97, so its expression can be silenced by NRSF (88). Here, we found that NRP-1 expression is lower in hippocampus of the DM + MCAO mice than in the MCAO mice. This reduction is in concert with the elevation of NRSF, because the NRSF knockdown resulted in significantly increased NRP-1 expression. Moreover, in the present study, we found that both VEGF and VEGFR2 levels change in consistent with the change pattern of NRP-1. Studies found that diabetic patients have decreased regional cerebral blood flow and immature brain microvasculature, with a decrease in VEGF (89). VEGF signaling acts on entire neurovascular cells, such as endothelial cells, neurons, and glia, and participates in chronic neurovascular regeneration. For example, synaptogenesis in hippocampal pyramidal neurons is triggered by NMDA receptor and VEGFR2 coactivation. Overexpression of VEGF also increases neurogenesis and improves hippocampal-dependent cognition (90–92). Since dysfunctional neurovascular network delays the functional recovery after brain injury, we conjectured that the reduction of NRP-1 and NRP-1/VEGF signaling is involved in NRSF-elevation-mediated neuronal injury and vasculature defects in diabetic ischemic mice.

Despite of these promising findings as discussed above, caution should be taken to extrapolate results to the female mice because only the male mice were used in the present study. Sex hormone is known to target cerebral vasculature and induce multiple functional changes. Several studies have shown that sex differences impact the outcome of both ischemia stroke and diabetes (93, 94). Males with type 2 diabetes were also associated with higher risk of ischemic stroke compared with female counterparts (95, 96). Therefore, considering potential confounding effect of sex differences on response to ischemia stroke and the drastic hormonal changes in adult female mice following estrous cycles, we controlled the sex differences by only using male mice in this study. Further studies are warranted in the future to investigate whether NRSF plays similar roles in female mice.

In the present study, the experimental paradigm is a little bit different between the Experiments 1 and 2. We performed the MCAO surgery 1 week after the hyperglycemia onset in the Experiment 1, whereas 1 month thereafter in the Experiment 2. As shown in the present study, both shorter and longer hyperglycemia duration similarly enhanced brain injury in the DM + MCAO mice. The shorter hyperglycemia duration has been considered as diabetes in many studies (97–99), in our study, we also observed severer neurobehavioral disabilities in these mice after the MCAO surgery; however, strictly speaking, it represents hyperglycemia rather than diabetes. Therefore, we performed MCAO under a relatively longer diabetes duration in the Experiment 2 for better understanding the role of NRSF in diabetic ischemic injury.

In conclusion, we demonstrated that the elevation of transcriptional factor NRSF leads to greater neuronal injury and brain microvasculature defects in diabetic ischemic hippocampus, which is possibly mediated *via* lessening its downstream NRP-1/VEGF signaling. Our data elucidated, to some extent, why diabetes exacerbates brain ischemic damage and cognition impairment, and put forward a new target candidate NRSF for the therapeutic practice of diabetic stroke.

## Data Availability Statement

The original contributions presented in the study are included in the article/Supplementary Material, further inquiries can be directed to the corresponding author/s.

## Ethics Statement

The animal study was reviewed and approved by the Institutional Animal Care and Use Committee of Shanghai Medical College of Fudan University.

## Author Contributions

J-CG, H-GZ, and YF designed the study. C-FH performed the experiments and analyzed and interpreted the data. W-JX, X-DX, J-TW, and X-RW contributed to the analysis of the results. All authors critically revised the manuscript and read and agreed to the published version of the manuscript.

## Funding

This work was supported by grants from the National Science Foundation of China (81671392 to J-CG and 81871098, 81571361 to H-GZ), Shanghai Municipal Science and Technology Major Project (No. 2018SHZDZX01), ZJ Lab, and Shanghai Center for Brain Science and Brain-Inspired Technology to J-CG, the Projects of Shanghai Health and Health Committee on Integration of Traditional Chinese and Western Medicine [ZY(2018-2020)-FWTX-3007, ZHYY-ZXYJHZX-201915] and Shanghai Municipal Key and Clinical Specialty (Geriatrics, No. shslczdzk02802) to H-GZ, and the Development Project of Shanghai Peak Disciplines Integrated Chinese and Western Medicine (20180101 to YF).

## Conflict of Interest

The authors declare that the research was conducted in the absence of any commercial or financial relationships that could be construed as a potential conflict of interest.

## Publisher's Note

All claims expressed in this article are solely those of the authors and do not necessarily represent those of their affiliated organizations, or those of the publisher, the editors and the reviewers. Any product that may be evaluated in this article, or claim that may be made by its manufacturer, is not guaranteed or endorsed by the publisher.
