# Supplementary material for: Knockdown of NRSF Alleviates Ischemic Brain Injury and Microvasculature Defects in Diabetic MCAO Mice
Source: Front Neurol. 2022 May 13;13:869220. doi: 10.3389/fneur.2022.869220 (PMC9136417; doi:10.3389/fneur.2022.869220)
Supplement: Supplementary file 1 [file Data_Sheet_1.PDF]

Supplementary Material

1. Supplementary Figures

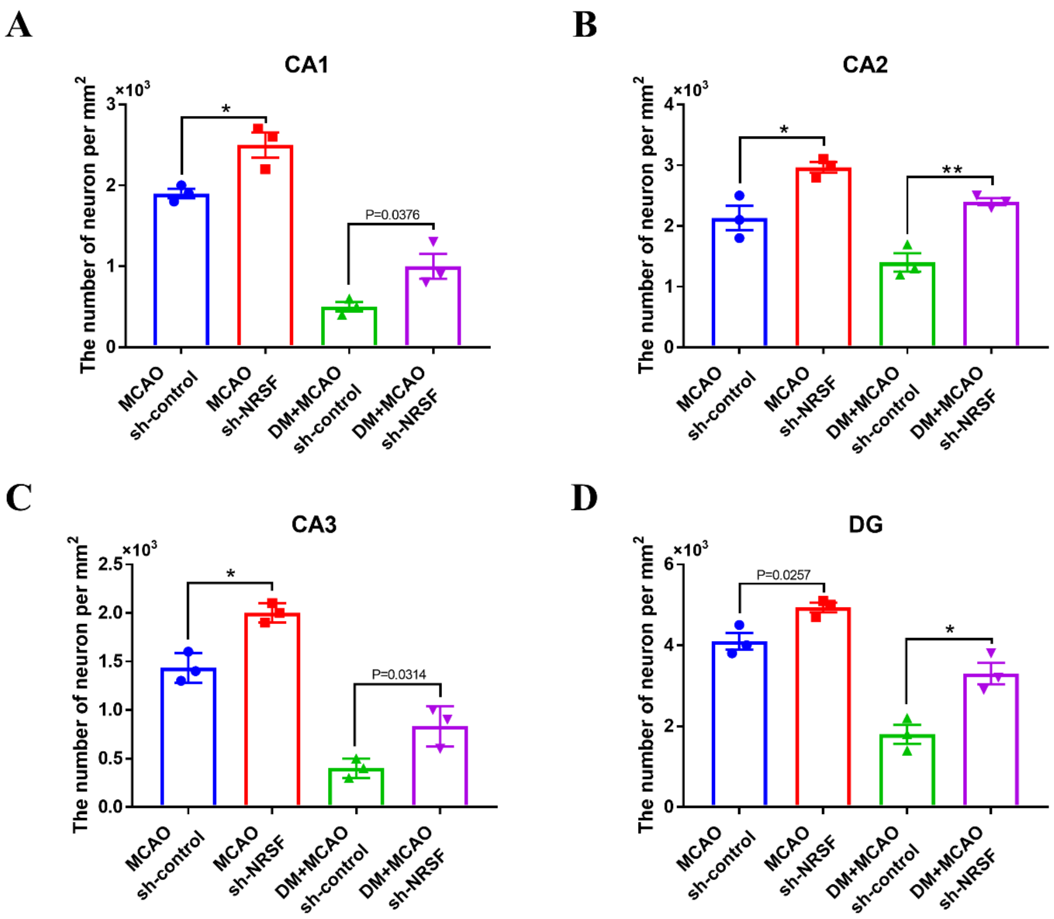

Supplementary Figure 1. The number of surviving neurons per mm^2 of CA1, CA2, CA3, DG in all groups. n = 3 for each group. Data were shown as mean ± SEM (Two-way ANOVA, Student's t-test). *P＜0.025, **P＜0.005.

2. Supplementary Tables

Table S1. Descriptive statistics of experiment in all groups

Experiment	Group	mean±SEM / median (interquartile range)	Passed normality test?
TTC	sham	0.0000±0.0000	Yes
	MCAO	0.1428±0.0089	Yes
	DM+MCAO	0.3276±0.0329	Yes
Neurobehavioral score	sham	0.00 (0.00, 0.00)	
	MCAO	2.50 (2.00, 3.75)	
	DM+MCAO	8.50 (6.50, 12.75)	
Rotarod	sham	0.9867±0.0222	Yes
	MCAO	0.8302±0.0331	Yes
	DM+MCAO	0.1199±0.0311	Yes
Grid walking	sham	6.6250±0.8004	Yes
	MCAO	18.0000±1.2820	Yes
	DM+MCAO	28.5000±2.2120	Yes
Y maze	sham	81.000±3.9210	Yes
	MCAO	65.8300±3.9410	Yes
	DM+MCAO	51.3000±3.9290	Yes
Numbers of GFAP+ cells	sham	6.50 (5.25, 8.50)	
	MCAO	16.50 (15.25, 20.00)	

	DM+MCAO	32.00 (28.75, 36.00)	
PDGFR-β+ cell coverage (%)	sham	87.7600±3.6140	Yes
	MCAO	53.4300±2.6800	Yes
	DM+MCAO	29.7000±4.4580	Yes
Vascular length	sham	1.0000±0.0429	Yes
	MCAO	0.6614±0.0574	Yes
	DM+MCAO	0.3357±0.0573	Yes
Vascular area	sham	1.0000±0.0525	Yes
	MCAO	0.6872±0.0379	Yes
	DM+MCAO	0.3907±0.0305	Yes
NRSF Integrated intensity	sham	1.0000±0.2661	Yes
	MCAO	7.0150±0.9017	Yes
	DM+MCAO	20.1100±2.8970	Yes
NRSF	sham	1.0000±0.1525	Yes
	MCAO	2.0220±0.3538	Yes
	DM+MCAO	3.8410±0.5903	Yes
HDAC1	sham	1.0000±0.1068	Yes
	MCAO	1.2940±0.2433	Yes
	DM+MCAO	1.4670±0.1060	Yes
mSin3A	sham	1.0000±0.0906	Yes
	MCAO	1.7230±0.1316	Yes
	DM+MCAO	2.5740±0.3080	Yes

β-TrCP	sham	1.0000±0.0824	Yes
	MCAO	0.7173±0.1326	Yes
	DM+MCAO	0.6605±0.1113	Yes
NRSF	MCAO sh-control	0.0427±0.0047	Yes
	MCAO sh-NRSF	0.0098±0.0015	Yes
	DM+MCAO sh-control	0.1635±0.0268	Yes
	DM+MCAO sh-NRSF	0.0458±0.0080	Yes
Surviving neurons per mm^2 of CA1	MCAO sh-control	2.0000±0.0577 ($\times 10^3$)	Yes
	MCAO sh-NRSF	2.5000±0.1528 ($\times 10^3$)	Yes
	DM+MCAO sh-control	0.5000±0.0577 ($\times 10^3$)	Yes
	DM+MCAO sh-NRSF	1.0000±0.1528 ($\times 10^3$)	Yes
Surviving neurons per mm^2 of CA2	MCAO sh-control	2.1330±0.2028 ($\times 10^3$)	Yes
	MCAO sh-NRSF	2.9670±0.0882 ($\times 10^3$)	Yes
	DM+MCAO sh-control	1.4000±0.1528 ($\times 10^3$)	Yes

	DM+MCAO sh-NRSF	2.4000±0.0577 (×10^3)	Yes
Surviving neurons per mm^2 of CA3	MCAO sh-control	1.4330±0.0882 (×10^3)	Yes
	MCAO sh-NRSF	2.0000±0.0577 (×10^3)	Yes
	DM+MCAO sh-control	0.4000±0.0577 (×10^3)	Yes
	DM+MCAO sh-NRSF	0.8333±0.1202 (×10^3)	Yes
Surviving neurons per mm^2 of DG	MCAO sh-control	4.1000±0.2082 (×10^3)	Yes
	MCAO sh-NRSF	4.9330±0.1202 (×10^3)	Yes
	DM+MCAO sh-control	1.8000±0.2309 (×10^3)	Yes
	DM+MCAO sh-NRSF	3.3000±0.2646 (×10^3)	Yes
Neurobehavioral score	MCAO sh-control	3.00 (2.00, 4.00)	
	MCAO sh-NRSF	3.00 (1.75, 3.25)	
	DM+MCAO sh-control	11.00 (8.75, 12.25)	
	DM+MCAO sh-NRSF	11.00 (8.50, 12.00)	
	MCAO	20.8700±0.9999	Yes

MW Swimming speed	sh-control		
	MCAO sh-NRSF	20.6800±0.3927	Yes
	DM+MCAO sh-control	20.5800±0.7196	Yes
	DM+MCAO sh-NRSF	21.6500±1.7830	Yes
MW Times of platform crossing	MCAO sh-control	2.00 (0.75, 2.25)	
	MCAO sh-NRSF	3.00 (2.00, 4.25)	
	DM+MCAO sh-control	0.50 (0.00, 1.00)	
	DM+MCAO sh-NRSF	2.00 (0.75, 2.25)	
MW Percentage of time in target	MCAO sh-control	25.6400±1.9000	Yes
	MCAO sh-NRSF	36.4000±3.7760	Yes
	DM+MCAO sh-control	19.2300±2.2600	Yes
	DM+MCAO sh-NRSF	27.6100±2.5860	Yes
Y maze	MCAO sh-control	64.3500±4.2780	Yes
	MCAO	81.1400±3.8450	Yes

	sh-NRSF		
	DM+MCAO sh-control	52.5100±2.5480	Yes
	DM+MCAO sh-NRSF	71.9800±5.1620	Yes
NRP-1	MCAO sh-control	0.5715±0.0868	Yes
	MCAO sh-NRSF	1.7240±0.2625	Yes
	DM+MCAO sh-control	0.1723±0.0177	Yes
	DM+MCAO sh-NRSF	0.8845±0.0718	Yes
VEGF	MCAO sh-control	194.6000±10.0500	Yes
	MCAO sh-NRSF	265.7000±8.7020	Yes
	DM+MCAO sh-control	130.4000±12.5500	Yes
	DM+MCAO sh-NRSF	204.8000±5.5260	Yes
VEGFR2	MCAO sh-control	213.3000±14.4800	Yes
	MCAO sh-NRSF	264.6000±8.0930	Yes
	DM+MCAO sh-control	154.6000±9.7890	Yes

	DM+MCAO sh-NRSF	224.5000±9.9440	Yes
HDAC1	MCAO sh-control	0.3691±0.0049	Yes
	MCAO sh-NRSF	0.3097±0.0046	Yes
	DM+MCAO sh-control	0.4689±0.0132	Yes
	DM+MCAO sh-NRSF	0.3823±0.0224	Yes

Table S2. Statistical results of experiment in all groups

Experiment	Group	Statistical tests	Adjusted P Value
TTC	sham vs. MCAO	Dunnett's multiple comparisons test	0.0039
	MCAO vs. DM+MCAO	Dunnett's multiple comparisons test	0.0010
Neurobehavioral score	sham vs. MCAO	Dunn's multiple comparisons test	0.0478
	MCAO vs. DM+MCAO	Dunn's multiple comparisons test	0.0415
Rotarod	sham vs. MCAO	Dunnett's multiple comparisons test	0.0020
	MCAO vs. DM+MCAO	Dunnett's multiple comparisons test	<0.0001
Grid walking	sham vs. MCAO	Dunnett's multiple comparisons test	<0.0001

	MCAO vs. DM+MCAO	Dunnett's multiple comparisons test	0.0002
Y maze	sham vs. MCAO	Dunnett's multiple comparisons test	0.0233
	MCAO vs. DM+MCAO	Dunnett's multiple comparisons test	0.0300
Numbers of GFAP+ cells	sham vs. DM+MCAO	Dunn's multiple comparisons test	0.0051
PDGFR-β+ cell coverage (%)	sham vs. MCAO	Dunnett's multiple comparisons test	0.0002
	MCAO vs. DM+MCAO	Dunnett's multiple comparisons test	0.0024
Vascular length	sham vs. MCAO	Dunnett's multiple comparisons test	0.0027
	MCAO vs. DM+MCAO	Dunnett's multiple comparisons test	0.0034
Vascular area	sham vs. MCAO	Dunnett's multiple comparisons test	0.0009
	MCAO vs. DM+MCAO	Dunnett's multiple comparisons test	0.0012
NRSF Integrated intensity	sham vs. DM+MCAO	Tukey's multiple comparisons test	<0.0001
	MCAO vs. DM+MCAO	Tukey's multiple comparisons test	0.0013
NRSF	sham vs. DM+MCAO	Tukey's multiple comparisons test	0.0021
	MCAO vs. DM+MCAO	Tukey's multiple comparisons test	0.0281
HDAC1	sham vs. DM+MCAO	Unpaired t test	0.0210
mSin3A	sham vs. DM+MCAO	Tukey's multiple comparisons test	0.0009

	MCAO vs. DM+MCAO	Tukey's multiple comparisons test	0.0359
β-TrCP	sham vs. DM+MCAO	Unpaired t test	0.0496
NRP-1	sham vs. DM+MCAO	Tukey's multiple comparisons test	0.0231
VEGF	sham vs. MCAO	Dunnett's multiple comparisons test	0.0360
	MCAO vs. DM+MCAO	Dunnett's multiple comparisons test	0.0380
VEGFR2	sham vs. MCAO	Dunnett's multiple comparisons test	0.0101
	MCAO vs. DM+MCAO	Dunnett's multiple comparisons test	0.0449
NRSF	Row Factor	Two-way ANOVA	0.0006
	MCAO sh-control vs. MCAO sh-NRSF	Unpaired t test	0.0026
	DM+MCAO sh-control vs. DM+MCAO sh-NRSF	Unpaired t test	0.0137
Surviving neurons per mm2 of CA1	Row Factor	Two-way ANOVA	<0.0001
	MCAO sh-control vs. MCAO sh-NRSF	Unpaired t test	0.0213
	DM+MCAO sh-control vs. DM+MCAO sh-NRSF	Unpaired t test	0.0376
Surviving neurons per mm2 of CA2	Row Factor	Two-way ANOVA	0.0015
	MCAO sh-control vs. MCAO sh-NRSF	Unpaired t test	0.0196

	DM+MCAO sh-control vs. DM+MCAO sh-NRSF	Unpaired t test	0.0036
Surviving neurons per mm2 of CA3	Row Factor	Two-way ANOVA	<0.0001
	MCAO sh-control vs.<br><br>MCAO sh-NRSF	Unpaired t test	0.0058
	DM+MCAO sh-control vs. DM+MCAO sh-NRSF	Unpaired t test	0.0314
Surviving neurons per mm2 of DG	Row Factor	Two-way ANOVA	<0.0001
	MCAO sh-control vs.<br><br>MCAO h-NRSF	Unpaired t test	0.0257
	DM+MCAO sh-control vs. DM+MCAO sh-NRSF	Unpaired t test	0.0129
Neurobehavioral score	MCAO sh-control vs.<br><br>MCAO sh-NRSF	Mann Whitney test	0.7662
	DM+MCAO sh-control vs. DM+MCAO sh-NRSF	Mann Whitney test	0.7251
MW Swimming speed	Row Factor	Two-way ANOVA	0.7591
	MCAO sh-control vs.<br><br>MCAO sh-NRSF	Unpaired t test	0.8649
	DM+MCAO sh-control vs. DM+MCAO sh-NRSF	Unpaired t test	0.5889
MW Times of platform crossing	MCAO sh-control vs.<br><br>MCAO sh-NRSF	Mann Whitney test	0.0544
	DM+MCAO sh-control vs. DM+MCAO sh-NRSF	Mann Whitney test	0.0544
	Row Factor	Two-way ANOVA	0.0113

MW Percentage of time in target	MCAO sh-control vs. MCAO sh-NRSF	Unpaired t test	0.0291
	DM+MCAO sh-control vs. DM+MCAO sh-NRSF	Unpaired t test	0.0349
Y maze	Row Factor	Two-way ANOVA	0.0178
	MCAO sh-control vs. MCAO sh-NRSF	Unpaired t test	0.0153
	DM+MCAO sh-control vs. DM+MCAO sh-NRSF	Unpaired t test	0.0070
NRP-1	Row Factor	Two-way ANOVA	0.0025
	MCAO sh-control vs. MCAO sh-NRSF	Unpaired t test	0.0141
	DM+MCAO sh-control vs. DM+MCAO sh-NRSF	Unpaired t test	0.0006
VEGF	Row Factor	Two-way ANOVA	<0.0001
	MCAO sh-control vs. MCAO sh-NRSF	Unpaired t test	0.0017
	DM+MCAO sh-control vs. DM+MCAO sh-NRSF	Unpaired t test	0.0016
VEGFR2	Row Factor	Two-way ANOVA	0.0007
	MCAO sh-control vs. MCAO sh-NRSF	Unpaired t test	0.0214
	DM+MCAO sh-control vs. DM+MCAO sh-NRSF	Unpaired t test	0.0024
HDAC1	Row Factor	Two-way ANOVA	0.0002

	MCAO sh-control vs.<br><br>MCAO sh-NRSF	Unpaired t test	0.0009
	DM+MCAO sh-control vs.<br>DM+MCAO sh-NRSF	Unpaired t test	0.0291
mSin3A	Row Factor	Two-way ANOVA	<0.0001
	MCAO sh-control vs.<br><br>MCAO sh-NRSF	Unpaired t test	0.0007
	DM+MCAO sh-control vs.<br>DM+MCAO sh-NRSF	Unpaired t test	0.0008
